# Characteristics and Future Direction of Tibialis Posterior Tendinopathy Research: A Scoping Review

**DOI:** 10.3390/medicina58121858

**Published:** 2022-12-16

**Authors:** Hye Chang Rhim, Ravi Dhawan, Ashley E. Gureck, Daniel E. Lieberman, David C. Nolan, Ramy Elshafey, Adam S. Tenforde

**Affiliations:** 1 Department of Physical Medicine and Rehabilitation, Harvard Medical School, Boston, MA 02115, USA; 2 Department of Epidemiology and Biostatistics, T.H. Chan School of Public Health, Harvard University, Boston, MA 02115, USA; 3 Department of Human Evolutionary Biology, Harvard University, Cambridge, MA 02138, USA; 4 Department of Physical Therapy, Movement, and Rehabilitation Science, Northeastern University, Boston, MA 02115, USA; 5 Department of Orthopedics & Rehabilitation, Tufts Medical Center, 800 Washington Street, Boston, MA 02111, USA

**Keywords:** tibialis posterior tendinopathy, posterior tibial tendon dysfunction, acquired flatfoot deformity, progressive collapsing foot deformity

## Abstract

*Background and Objectives*: Tibialis posterior tendon pathologies have been traditionally categorized into different stages of posterior tibial tendon dysfunction (PTTD), or adult acquired flatfoot deformity (AAFD), and more recently to progressive collapsing foot deformity (PCFD). The purpose of this scoping review is to synthesize and characterize literature on early stages of PTTD (previously known as Stage I and II), which we will describe as tibialis posterior tendinopathy (TPT). We aim to identify what is known about TPT, identify gaps in knowledge on the topics of TPT, and propose future research direction. *Materials and Methods*: We included 44 studies and categorized them into epidemiology, diagnosis, evaluation, biomechanics outcome measure, imaging, and nonsurgical treatment. *Results*: A majority of studies (86.4%, 38 of 44 studies) recruited patients with mean or median ages greater than 40. For studies that reported body mass index (BMI) of the patients, 81.5% had mean or median BMI meeting criteria for being overweight. All but two papers described study populations as predominantly or entirely female gender. Biomechanical studies characterized findings associated with TPT to include increased forefoot abduction and rearfoot eversion during gait cycle, weak hip and ankle performance, and poor balance. Research on non-surgical treatment focused on orthotics with evidence mostly limited to observational studies. The optimal exercise regimen for the management of TPT remains unclear due to the limited number of high-quality studies. *Conclusions*: More epidemiological studies from diverse patient populations are necessary to better understand prevalence, incidence, and risk factors for TPT. The lack of high-quality studies investigating nonsurgical treatment options is concerning because, regardless of coexisting foot deformity, the initial treatment for TPT is typically conservative. Additional studies comparing various exercise programs may help identify optimal exercise therapy, and investigation into further nonsurgical treatments is needed to optimize the management for TPT.

## 1. Introduction

The tibialis posterior muscle is located within the deep posterior compartment of the leg, arising from the interosseous membrane and the adjacent fibula and tibia. In the distal third of the leg, the musculotendinous junction is formed, and the tendon passes behind the medial malleolus within a synovial sheath, beneath the flexor retinaculum. The tibialis posterior tendon inserts primarily onto the navicular tuberosity with multiple divisions attaching to the cuneiforms and bases of the second, third, and fourth metatarsals. The location of the tibialis posterior tendon relative to the axes of subtalar and ankle joints aids in inversion and plantarflexion, and the multiple insertion sites act to stabilize the medial longitudinal arch (MLA) of the foot [1,2,3]. A previous study showed that the tibialis posterior tendon serves to buffer the stretch of muscle fascicles during early stance and to facilitate the efficiency of the tibialis posterior through absorption and return of elastic strain energy [4]. Understanding the overall role of the tibialis posterior for foot and ankle function helps clinicians understand pathology and formulate strategies for non-operative treatment of tibialis posterior tendinopathy (TPT), especially in early stages of presentation.

Historically, the first case of TPT was reported by Kulowski in 1936 who described a case of posterior tendon tenosynovitis. This case was followed by several researchers who performed tenosynovectomies and debridement of the diseased tendon in the 1950–60s [5,6]. Since then, the importance of this tendon has been implicated toward the integrity of the medial and transverse arch of the foot, and conversely, progressive arch collapse has been associated with PTTD [7]. In 1989, Johnson and Strom laid out the first framework for understanding and categorizing different stages of posterior tibial tendon dysfunction (PTTD) [8]. According to their classification, Stage I is characterized with mild swelling, tenderness along the tibialis posterior tendon, mild weakness in heel-rise test while Stage II with moderate swelling, tenderness along the tibialis posterior tendon, marked weakness in heel-rise test, “too many toes” sign, and flexible foot deformity. In Stage III, marked ten-derness along the tibialis posterior tendon, marked weakness in heel-rise test, “too many toes” sign, and fixed foot deformity are present [8]. Several researchers [9,10,11,12] have subsequently expanded and redefined these classifications to understand the pathology of PTTD better and guide treatment decisions. At the same time, researchers began to use the term “adult acquired flatfoot deformity” (AAFD) interchangeably with PTTD even though these terms may have certain nuances of meaning. Ross et al. in 2017 posed a question whether PTTD and AAFD are the same in their systematic review and synthesized all articles with clear inclusion/diagnostic criteria for PTTD or AAFD [13]. While articles that used the term PTTD tended to describe both tendon pathology and foot deformities, articles that used the term AAFD focused more on structural deformity. 

In recognition that nomenclature for AAFD is confusing and that the tibialis posterior tendon pathology is not the only cause of AAFD, a group of experts recently proposed using the term “Progressive Collapsing Foot Deformity” (PCFD) and implementing a classification scheme using criteria based on the type, location, and flexibility of the deformity [14]. In this classification, the description of tibialis posterior tendon pathology which composes of Johnson and Strom Stage I and part of Stage II is no longer included. Perhaps this new classification may now allow both clinicians and researchers to focus on tibialis posterior tendon pathology as its own entity. In fact, the description of tibialis posterior tendon pathology including swelling and/or pain along the tendon along with difficulty loading the tendon during heel rise or inversion aligns with the consensus definition of tendinopathy [15,16,17]. Then, another way of understanding what was previously described as Stage I and II would be viewing them as tibialis posterior tendinopathy (TPT), but this term has been less frequently used compared to AAFD or PTTD [18]. Furthermore, while conservative management has been traditionally recommended for early stages of PTTD (i.e., TPT), it seems that overwhelming body of research has focused on surgical treatment [19,20]. 

In this scoping review, we aim to summarize the characteristics and results of studies on TPT with a focus on non-operative treatment since studies on surgical treatment seemed to outnumber those on nonsurgical treatment options. Our review highlights gaps in knowledge on the topics of TPT and proposes future research directions to advance understanding the biomechanics and effective management of this condition. 

## 2. Materials and Methods

### 2.1. Search Strategy and Selection Criteria

Authors performed a search using PubMed (Medline), Embase, and Web of Science from database inception through 1 December 2021. Search terms aimed to capture the following terms using the AND or OR commands: posterior tendinopathy, posterior tibial tendon dysfunction, posterior tibial tendon insufficiency, tibialis posterior tendonitis, tibialis posterior tendinosis, tibialis posterior tenosynovitis, acquired adult flatfoot deformity, pes planus, pes planovalgus, and progressive collapsing foot deformity. Secondary articles were identified by reviewing the reference lists of relevant review articles. Inclusion criteria were original studies investigating adult patients with TPT, PTTD/AAFD Stage I and II based on Johnson and Strom classifications, or clinical signs of TPT including difficulty with inversion and/or single leg heel raise with pain and/or swelling along the tendon. Articles selected were written in English or could be translated to English. Exclusion criteria were studies including the following: (1) traumatic tibialis posterior tendon ruptures; (2) Stage III or IV PTTD; (3) PCFD; (4) no description of stages or specific clinical signs of TPT; (5) patient populations with other systematic conditions or foot pathologies (e.g., symptomatic navicular bone, rheumatoid arthritis, fracture, severe osteoarthritis, etc.); (6) flatfoot of other or unknown etiologies; (7) surgical techniques or indications; (8) cadaveric experiments; (9) molecular/genetic/histological research; (10) simulation (finite element study); (11) reconstructed or other imaging studies to focus on foot structure or alignment rather than tibialis posterior pathology. Articles that were published or recruited patients prior to Johnson and Strom classification were excluded because of unclear pathology and difficulty of access. We also excluded case reports, reviews, conference proceedings, abstracts, opinions/editorials, protocols, and consensus statements (Table 1).

### 2.2. Data Extraction

Data extraction was independently conducted by two authors (HCR and RD). Each author manually extracted characteristics such as study topic, study design, population and inclusion/exclusion criteria, intervention, comparator, outcome measures, and follow-up period. Given the scoping nature of this review, quality assessment such as risk of bias was not performed [21,22]. 

## 3. Results

### 3.1. Search Results

**Figure 1:**
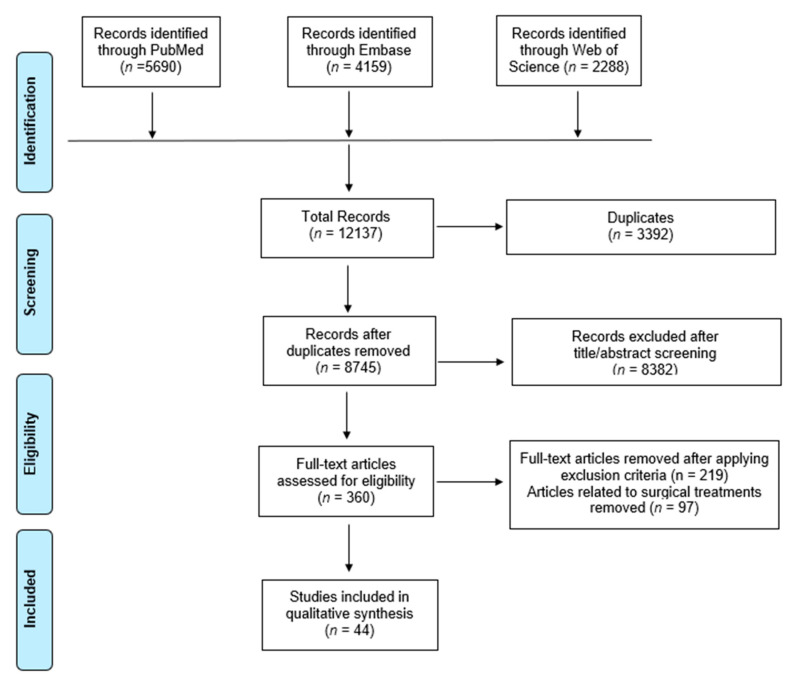
Flowchart showing selection of articles pertaining to tibialis posterior tendinopathy for scoping review and qualitative analysis.

After applying the search terms in each database, a total of 12,137 studies were initially identified. After removing 3392 duplicates, 8745 studies were screened. After screening abstracts, 8382 studies were found to be irrelevant, and 360 studies were eligible for full text review. Applying inclusion and exclusion criteria including removal of studies on surgical treatment (*n* = 97), 44 studies were included in this review (Figure 1). Topics related were summarized and analyzed on epidemiology, diagnosis, evaluation, biomechanics, outcome measure, imaging, and nonsurgical treatment (Table 2).

### 3.2. General Characteristics and Epidemiology

A total of 3 studies were on epidemiology; 19 studies were on diagnosis, evaluation, and biomechanics; 10 studies were on imaging; 12 studies were on nonsurgical treatment, and one study was on outcome measure. A majority of studies (86.4%: 38 of 44 studies) recruited patients with mean or median ages greater than 40. For studies that reported body mass index (BMI) of the patients, 81.5% had mean or median BMI meeting criteria for being overweight with values greater than 25 kg/m^2^. The range in mean or median symptom durations was from 3–40 months. All but two papers described study populations as predominantly or entirely female gender. One study recruited runners as study participants [48]. Patient-reported outcomes including Foot Functional Index (FFI), American Orthopaedic Foot and Ankle Society (AOFAS), and VAS were commonly used while foot orthotic use, muscle strength, patient satisfaction, distance of ambulation, 5 min walking test, range of motion, physical activity level, radiography, tendon morphology on ultrasound, single limb heel rise, FAAM were also measured across the studies. 

Three studies described the prevalence and anthropometric factors associated with TPT. The prevalence of TPT in England was 3.3% in women over the age of 40 [41]. In the Korean population, patients with TPT tended to have higher BMI, but when compared to the mean BMI of an age-matched population, there was no significant difference in patients between 21 and 70 years of age [45]. In a case–control study, the prevalence of leg length discrepancy (LLD), and mean absolute and relative LLD values were significantly higher in patients with TPT, suggesting that LLD may be a risk factor for the development of TPT [53]. 

### 3.3. Diagnosis, Evaluation, and Biomechanics

Cooper et al. studied 15 patients (17 ankles) who completed magnetic resonance imaging (MRI) for clinically suspected TPT and received local anesthetic injection into the posterior tibial tendon sheath. In all patients, pain was completely relieved without any complication, confirming the clinical impression that the tibialis posterior was the pain generator. Fifteen ankles showed abnormally increased fluid signals within the sheath on MRI while two ankles had negative MRI findings with subsequent surgery revealing inflammatory changes. Collective results suggested local anesthetic tendon sheath injection may aid in diagnosing TPT in clinically suspicious patients who have negative MRI findings [32].

Two studies investigated foot and ankle kinematics during single-limb heel-rise test [52] and bilateral heel rise test [39]. During the single-limb heel-rise test, patients demonstrated lower heel-rise height compared to the younger control group. Compared with the older control group, those with TPT displayed higher degrees of first metatarsal dorsiflexion, lower ankle plantar flexion, higher subtalar eversion, lower ankle excursion, and lower first metatarsal excursion [52]. During the bilateral heel rise test, patients demonstrated less hallux dorsiflexion but both had greater ankle plantar flexion and first metatarsal dorsiflexion compared to the controls but similar hindfoot inversion between the groups, suggesting that MLA kinematics may be more sensitive than hindfoot kinematics during a bilateral heel rise test [39].

One study evaluated deep compartment muscle strength on patients with TPT, and two studies investigated the effect of deep compartment muscle strength on foot kinematics and total and distributed plantar loading. Houck et al. reported patients with TPT had both reduced subtalar inversion and forefoot adduction strength by 20–30% compared to the controls [35]. Neville et al. divided 30 patients into strong and weak groups based on the deep posterior compartment strength and showed that as a group, patients with TPT demonstrated significantly greater hindfoot eversion compared to the controls. Within the weaker group, greater hindfoot eversion was measured compared to the strong group. The patients with weak strength also showed greater forefoot abduction than the controls across loading response, midstance, and terminal stance, and a greater MLA angle (lower MLA) than controls at terminal stance [46]. A separate report using the same patient population by Neville et al. found that patients with TPT demonstrated altered total and distributed loading during the end of stance suggesting impaired capability to achieve normal push-off mechanics with altered distributed loading further influenced by weak deep compartment muscle strength [51]. Kulig et al. investigated ankle and hip muscle performance on 17 female patients and identified weaker ankle and hip function bilaterally than the controls with significantly fewer single-limb heel raises and repeated sagittal and frontal plane non-weightbearing leg raises and lower hip extensor and abductor torques [47].

Kulig et al. assessed the balance performance of 19 female patients and found that the success rate of the unipedal standing balance tests was significantly reduced in patients with TPT compared to the controls and correlated with the number of single limb heel raises. Furthermore, these patients displayed increased anterior-posterior center of pressure displacement and a strong trend of increased medial-lateral center of pressure displacement while completing the unipedal standing balance tests [56].

Four studies evaluated biomechanical effects of foot and ankle orthosis for patients with TPT. Chicoine et al. found that hindfoot eversion angles and ankle inversion moments were decreased while ankle eversion moments were increased with custom foot orthosis compared to the shoes only and prefabricated foot orthosis among 14 patients. However, an increased knee abduction moment was induced in neutral custom foot orthosis and custom varus foot orthosis conditions compared to the shoes only. There was no change in hip kinematics and kinetics among the conditions [63]. Neville et al. in 2009 tested the AirLift PTTD brace with differing airbladder inflation on 10 female patients and found that on average, this brace was successful in decreasing the amount of hindfoot eversion [44]. Neville et al. in 2012 compared shoe only, shoe with a custom solid AFO (Arizona Co., Mesa, AZ, USA), and shoe with a custom articulated AFO (Arizona Co., Mesa, AZ, USA), and shoe with an off-the shelf AFO (AirLift) on 14 patients. The study showed that custom orthoses increased hindfoot inversion and forefoot plantarflexion compared to the shoe only condition while the AirLift caused forefoot plantarflexion without changing hindfoot motion. None of the orthoses corrected forefoot abduction compared to walking with shoes without orthotics [49] Neville et al. reported greater change in forefoot adduction using AFO with a lateral extension compared to the standard AFO and shoe-only conditions across all phases of stance in 15 patients and increased hindfoot inversion during both loading response and terminal stance phases [57].

Rabbito et al. investigated biomechanical and clinical factors in 12 runners with TPT. The authors found that runners with TPT had greater and prolonged peak rearfoot eversion angle during gait along with significantly lower seated arch height index, but there were no significant differences in standing arch height index, arch rigidity index, ankle invertor strength, or peak medial longitudinal arch compared to non-injured runners [48].

Ross et al. attempted to determine the reliability of common clinical tests for TPT, pain on palpation of the tendon, swelling around the tendon, pain/weakness with TP contraction, and pain or inability to perform single limb heel raise and investigate their relationship with ultrasound findings. The authors found that the single limb heel raise was the most reliable test for TPT; however, patients who were positive with the single limb heel raise test did uniformly have structural changes under ultrasound [18].

Heng et al. compared first ray (first metatarsocuneiform joint) mobility in 16 flatfooted individuals exhibiting TPT and 16 flatfooted controls. An upward force underneath the first metatarsal head was applied with the lesser metatarsals held in place to quantify maximum dorsal displacement of the first ray. The authors found that there were no significant differences in first ray displacement between the groups [58].

### 3.4. Imaging

Excluding imaging studies that investigated foot structure and alignment, 10 studies evaluated tibialis posterior and its tendon on patients with clinical signs or diagnosis of TPT using ultrasound, MRI, and plain radiographs. Chen et al. observed sonographic differences with significantly greater diameters of inner wall of tendon sheath and tendon size in symptomatic TPT compared to those of asymptomatic tendons [24], and Hsu et al. demonstrated that ultrasound was useful in identifying tenosynovitis and complete rupture of tibialis posterior tendon when compared with the operative findings and MRI [25].

Lim et al. identified secondary MRI findings that may aid in diagnosis of TPT such as posterior tibialis sheath fluid, distal tibial spur located anterior to the tibialis posterior tendon, and unroofing of the talus. While a wide range of sensitivities (32–92%) and a moderate range of specificities (54–87%) were reported, tibial spurring and unroofing of the talus had high specificities of 89–93% and 78–100%, respectively [26]. Gonzalez et al. found greater posterior tibialis sheath fluid volume on the MRI in patients with TPT compared to other causes of medial ankle pain and asymptomatic controls [59]. Wacker et al. found atrophy of the tibialis posterior muscle in all patients compared to normal legs and replacement of the muscle by fatty infiltration in patients with a complete rupture of PTT as well as compensatory hypertrophy of the flexor digitorum longus [29]. Park et al. suggested that the PTT cross-sectional area may be a more valid predictor than thickness on MRI for the diagnosis of TPT [61]. 

Premkumar et al. described characteristics of TPT including enhancement of the tendon on MRI and increased anteroposterior diameter and inhomogeneity of the tendon on both MRI and ultrasound. When compared with MRI, ultrasound showed the sensitivity of 80% and specificity of 90% for diagnosing TPT [27]. Perry et al. demonstrated that while MRI tendon and peritendon enhancement and ultrasound tendon and peritendon flow were both associated with increasing pain intensity during clinical examination, MRI was more sensitive in detecting posterior tibialis tendon tear [28]. However, Arnolder et al. found that high-resolution ultrasound may be slightly more accurate than 3T MRI for detecting TPT [54]. 

Kwon et al. showed that PTT integrity can be screened with weight-bearing anteroposterior foot radiography with findings of wavy pattern or an irregular margin for the tendon shadow is observed if the tendon thickness was different compared to the contralateral side (>2 mm difference at the medial end of the talar head) suggestive of TPT [60].

### 3.5. Nonsurgical Treatment

A total of 19 studies were identified after screening for nonsurgical treatment of PTTD, but four studies included stages from I-III [66,67,68,69]; one study included stages I-IV [70]; one study was a published research protocol [71]; and one study did not specify stages [72]. Among 12 studies that met the inclusion criteria (Table 3), four studies were randomized clinic trials [36,43,55,65] and the remaining eight studies were observational [23,30,37,38,42,45,62,64]. 

Three studies investigated the effect of orthosis [23,37,38], and two studies assessed the effect of exercise [36,64], while five studies aimed to evaluate the combined effect of exercise and orthosis [30,42,45,55,71]. One randomized trial compared the effects of low level laser therapy (LLLT) and orthosis [65], and one case series evaluated combined extracorporeal shockwave therapy (ESWT) with exercise [62].

Chao et al. evaluated molded ankle-foot orthosis (MAFO) and a University of California Biomechanics Laboratory (UCBL) shoe insert with medial posting depending on foot deformity and obesity. Patients with obesity and more severe deformity received MAFO while the others received UCBL. The average duration of orthosis use was 14.9 months. The study found that 67% of patients had good to excellent response based on pain, function, and satisfaction. The remaining 33% discontinued use of the orthosis at follow-up evaluation due to concurrent medical conditions, discomfort, and poor response resulting in surgery [23]. Krause et al. used the custom molded foot orthosis “(shell brace)” on 18 patients who had symptoms present for a mean of 29 months and treated with insoles, physical therapy, and NSAIDs. 15 patients reported pain relief within an average of 2.8 weeks from the initiation of the orthosis. At the latest follow-up period (mean 61.4 months), the AOFAS ankle hindfoot score significantly improved from a mean of 56 points to a mean of 82 points. Fifteen patients were satisfied with the brace’s comfort and experienced improvement of mobility. Three patients underwent a clinical progression and radiographic increase of their deformity. Development of calluses occurred in three patients [37]. Lin et al. evaluated Double Upright Ankle Foot Orthosis (DUAFO) on 32 patients with a minimum follow-up of 7 years. The mean duration of initial bracing was 14.9 months. The study found that 69.7% of the patients were brace-free and able to avoid surgery at an average follow-up of 8.6 years with significant improvement in AOFAS hindfoot score, Foot Functional Index (FFI), and VAS pain score [38].

Four studies performed gait analysis to investigate foot and ankle kinematics and kinetics in patients with TPT compared to healthy controls. Neville et al. showed greater posterior tibial length relative to the subtalar neutral position across all phases of stance in 17 patients with TPT compared to the control group [34]. Ringleb et al. observed limited hindfoot eversion and increased midfoot external rotation during the heel rocker (first rocker) and forefoot rocker (third rocker) as well as compensatory muscle activity in the peroneal muscles, tibialis anterior, and gastrocnemius in five patients [33]. Tome et al. identified greater rearfoot eversion and medial longitudinal arch (MLA) angles during loading response and greater MLA angle and forefoot abduction angle during pre-swing in 14 patients [31]. Houck et al. found in 30 patients greater ankle plantarflexion prior to or at stance, and both greater hindfoot eversion and first metatarsal dorsiflexion across stance [40].

Two studies investigated the effect of exercise on the clinical outcomes of TPT. Jeong et al. conducted a randomized trial comparing a group of 7 females who received 6-week stretching and strengthening program with a control group comprised of 5 females (mean 53.2 years old) that described differences in muscle function, range of motion, pain, and gait. The authors reported the exercise group demonstrated significant reduction in pain, improved dorsiflexion range of motion, and increased dorsiflexion and plantarflexion powers compared with controls [36]. Kim et al. evaluated the effect of a 4-week short foot exercise (SFE) on pain and changes in ankle joint kinematics and kinetics, and extrinsic activation of tibialis anterior and fibularis longus muscles. The authors noticed changes in muscle activation patterns for tibialis anterior and fibularis longus muscles, but these changes could not influence the ankle joint mechanics or structural deformity. The VAS pain score did not significantly improve after 1 month of the SFE program [64].

Five studies investigated the combined effects of orthosis and a home exercise program. Alvarez et al. used articulated AFO or foot orthosis along with strengthening exercises for tibialis posterior, peroneal muscles, tibialis anterior, and gastroc-soleus composed of isokinetic exercises, an exercise band, heel rises, and toe walking on 47 patients with the median symptom duration of 135 days. At a median period of 4 months, 83% patients had successful outcomes defined as no more than 10% strength deficit compared to the uninjured tibialis posterior, the ability to accomplish 50 single-support heel rises with none or minimal pain, the ability to walk 100 feet on the toes with none or minimal pain, and the ability to perform 200 repetitions of the home exercises for each muscle group. Five patients in this cohort elected surgery [30]. Chung et al. used a six-week program that modified the protocol by Alvarez et al. in 42 female patients. At the mean follow-up of 20 months, VAS pain scores decreased across the cohort, and most patients with difficulty performing single heel rise (28 of 39 patients) reported improvements. Five patients elected to complete surgical treatment. Kulig et al. evaluated the effect of a 10-week eccentric tibialis posterior exercises along with calf stretches and orthosis on 10 patients. At 3 months and at 6 months, the FFI scores improved, and the number of single heel raises significantly increased on the involved side at 3 months. Despite these symptomatic and functional improvements, tendon morphology and vascularization on Doppler ultrasound remained abnormal at 6 months [42]. In a randomized controlled trial, Kulig et al. investigated the effect of resistance exercise and orthosis. 36 patients were randomly assigned to orthosis and stretching group (O group), the combination with concentric progressive resistive exercise (OC group), and combination with eccentric progressive resistive exercise (OE group). While all three interventions improved the FFI scores at three months, the OE group showed the most improvement and least gain observed in the O group. No differences were observed between the OE group and OC group [43]. Houck et al. also conducted a randomized controlled trial comparing 17 patients undergoing orthosis with stretching and 19 patients undergoing orthosis with stretching and eccentric and concentric exercises. Both groups significantly demonstrated improvement in pain and function measured by FFI and the Short Musculoskeletal Function Assessment over the 12 weeks. However, there was minimal difference in pain and function and no difference in isometric deep posterior compartment strength between the groups [55].

One randomized trial compared low level light therapy (LLLT) with insoles. In this study, 26 patients were assigned with insoles and required to use their insoles daily for 8 weeks while 26 patients with the mean age of 22 in the LLLT group received a treatment dose of 0.7–7 (j/cm^2^) gallium aluminum arsenide laser bilaterally at a wavelength of 850 nm for 3 days a week for a total of 14 sessions. Significant improvements were observed in pain and function in both groups after the treatments based on the FFI scores and International Physical Activity Questionnaire (IPAQ). At 9 months, insoles appeared more effective. Furthermore, both treatments did not have clinically important effects on invertor and evertor muscle strengths [65].

One case series evaluated the combined effect of ESWT and exercise. Ten patients who did not respond to previous physical therapy and/or orthosis were treated with a radial shockwave over a minimum of 3 weekly sessions combined with a foot core exercise program. At the median follow-up of 4 months, clinically important differences in the Foot and Ankle Ability Measure (FAAM) were achieved in 90% and 80% of patients for activities of daily living (ADL) and sport subscales, respectively, without any adverse event [62]. 

### 3.6. Outcome Measures

Mani et al. were concerned that many of the numerous instruments used in the foot and ankle literature on TPT were unvalidated or unresponsive. Therefore, the authors aimed to validate the Foot and Ankle Outcome Score (FAOS) for use in evaluating patients with TPT. The FAOS is self-administered survey composed of 42 items within 5 subscales: pain, other symptoms, ADLs, Sports and Recreational Activities, and Foot and Ankle Related Quality of Life. The authors found that the FAOS demonstrated acceptable construct and content validity, reliability, and responsiveness to be used as an outcome measure [50].

## 4. Discussion

The purpose of this scoping review was to identify studies that investigated tibialis posterior tendon pathologies describing TPT, to analyze patient characteristics and associated risk factors and treatment outcomes for non-operative treatment, and to propose future research directions.

### 4.1. Epidemiology and Risk Factors

The limited studies described females with higher BMI and age greater than 40 and suggested an incomplete understanding regarding the prevalence or incidence of TPT in most ages, geography, and male gender. One study described prevalence of 3.3% in women over the age of 40 in England [41]. One study was limited to athletes and evaluated runners and detected limited biomechanical differences in those with TPT [48]. Given that TPT may result from land-based sports including running [73] and dancing [74], further investigations characterizing epidemiology, risk factors, and treatment response from young and athletic populations, as well as more males, may help both understand the prevalence of this condition as well as establish treatment and prevention strategies.

### 4.2. Clinical Evaluation

Single limb heel raise may be the most reliable clinical test for TPT [15]. For imaging, ultrasound may be the best initial imaging tool to aid in diagnosis if clinical examination is ambiguous [27,54]. Studies included in this review suggest patients with TPT may have high BMI, LLD, weak hip and ankle muscle performance, and poor balance. Previous studies have observed excessive eversion of the rearfoot in the longer limb [75], resulting in the foot bearing a greater load overall and more of this load shifting to the forefoot [76] while in the shorter leg there may be compensatory toe walking [77] with a prolonged propulsive phase of the gait cycle [78]. Studies on gait-analysis showed increased forefoot abduction and rearfoot eversion in patients with TPT, which is consistent with the result of a recent systematic review on gait alterations in patients PTTD [79], and the possibility of correcting these alterations with an orthosis. However, impairments outside the foot and ankle are important to address given impairments noted in more proximal muscle groups.

While FFI, VAS, and AOFAS scores were commonly used outcome measures across the studies, the only validated outcome measure specific for TPT was FAOS. If the validated outcome measure FAOS cannot be adopted more widely, the more commonly used scales such as FFI or AOFAS may need to be validated for TPT and allow for comparative outcomes across studies to determine treatment effectiveness.

### 4.3. Non-Surgical Treatment

Our search found that research related to surgical treatment vastly outnumbered nonsurgical treatment (97 articles versus 12 articles). Orthoses were the most studied intervention with beneficial effects lasting up to 7 years [38]. However, the evidence for orthosis efficacy is limited to largely descriptive prospective observational cohorts that assign orthotics to the full patient population. Our review identified four RCTs evaluating treatment including three of them investigating exercise treatment and the other evaluating LLLT. Exercise treatment had benefits in patients with TPT, consistent with the systematic review by Ross et al. which concluded from three RCTs [36,43,55] that strengthening exercises, especially eccentric exercises, may help improve pain and foot function [19]. However, the optimal exercise regimen and duration for TPT remains unclear due to the limited number of high-quality studies. Based on the findings from other tendinopathies, the duration of exercise program should aim for 12 weeks and both concentric (heavy-slow resistance) and eccentric loading may be beneficial. Furthermore, since a tight gastroc-soleus complex can lead to a greater load on tibialis posterior, programs integrating stretching of the gastroc-soelus complex as well as tibialis posterior strengthening would be important to consider. While Kulig et al. noticed that tendon morphology and vascularization on Doppler ultrasound remained abnormal at 6 months despite symptomatic and functional improvements with eccentric exercise program [42], tendon remodeling has been described to continue for up to 12 months [80].

More recently, foot core exercises have been implemented in patients with TPT [62,64]. Kim et al. found that 4-week foot core exercises may change muscle activation patterns for the tibialis anterior and fibularis longus muscles, but pain measured by VAS did not significantly change after 1 month [64]. The duration of the intervention might have been too short to elicit a positive effect on pain and function. In another study by Robinson et al., the majority of patients achieved clinically important differences in the FAAM scores using a combined foot core exercise program and ESWT treatment; the relative effects of the exercise program cannot be determined. Given these results as well as weak ankle performance and hip abduction strength deficit noted in patients with TPT [47], additional studies comparing diverse exercise regimens including strengthening of foot intrinsic muscles and proximal hip strength would help identify optimal exercise therapy.

Compared to more common foot and ankle conditions such as Achilles tendinopathy, (AT) or plantar fasciitis (PF) with more diverse nonsurgical treatment options with high level evidence [81,82], the number of studies investigating nonoperative treatment options for TPT besides exercise and orthoses is lacking. It is also noteworthy that there has been no study evaluating biologics such as platelet rich plasma injections, which are commonly used in AT or PF. More recently, LLLT and ESWT have been tested, but the efficacy of LLLT was limited compared to insole and the evidence for ESWT is poor. Given the degenerative tendon pathology identified in patients with TPT, interventions that fall into regenerative medicine categories including PRP and ESWT may be worth further exploration to determine clinical effectiveness.

### 4.4. Strength and Limitations

The strengths of our review include identifying studies that are related to TPT, organizing them according to different topics, and proposing future research directions to optimize the management of this condition. While our initial goal was to include all studies related to TPT, we decided to focus on nonsurgical treatments because the number of surgical studies (*n* = 97) outnumbered nonsurgical studies (*n* = 12), and therefore, evaluating studies on nonsurgical treatments would be more appropriate for a scoping review [21]. Although we advocate for more research on nonsurgical treatments, surgery remains an important treatment option when conservative measures fail. Furthermore, we excluded studies with patients on concurrent systematic conditions such as rheumatoid arthritis or other foot pathologies in an attempt to isolate tibialis tendon pathologies. Thus, the findings of this review may not be generalized to patients with such concurrent conditions. Lastly, we excluded other studies related to genetics, histology, cadaver, simulation, and reconstruction imaging. It is important to note that these studies may help clinicians further understand TPT pathology and formulate treatment strategies.

## 5. Conclusions

This scoping review summarizes and characterizes available research on TPT and identified topics on epidemiology, diagnosis, evaluation, biomechanics, imaging, nonsurgical treatment, and outcome measure. We conclude that more epidemiological studies from diverse patient populations are necessary to better understand prevalence, incidence, and risk factors. In addition, studies on surgical treatment currently outnumber those on nonsurgical treatment options, most likely due to the association of TPT with foot deformity now termed PCFD. The lack of high-quality studies investigating nonsurgical treatment options is a problem because, regardless of coexisting foot deformity, the initial treatment for TPT should be conservative. The mechanism for how tibialis posterior functions at different stages of TPT may help understand key function and how other variables such as footwear influence function. Exercise treatments need to be further studied and quantified for clinical outcomes, and interventions to target tendon remodeling may help with management of this condition.

## Figures and Tables

**Table 1:** Inclusion and exclusion criteria for study selection.

Inclusion Criteria	Exclusion Criteria
Studies that investigated adult patients with (1) TPT or (2) PTTD/AAFD Stage I and II or (3) clinical signs of TPT including difficulty with inversion and/or single leg heel raise with pain and/or swelling along the tendon	Case reports, reviews, conference proceedings, abstracts, opinion/editorials, protocols, and consensus statements; Studies that investigated patients with (1) traumatic tibialis posterior tendon ruptures (2) PTTD Stage III or IV (3) PCFD (4) No description of stages or specific clinical signs of TPT (5) concurrent systematic conditions or foot pathologies (6) flatfoot of other or unknown etiologies; Studies related to (7) surgical techniques or indications (8) cadaveric experiments (9) molecular/genetic/histologic research (10) simulation (11) reconstruction or other imaging focusing on foot structure or alignment

Abbreviations: PTTD, posterior tibial tendon dysfunction; PCFD, progressive collapsing foot deformity; TPT, tibialis posterior tendinopathy.

**Table 2:** Characteristics of included studies.

Author (Year)	Type of Studies	Age	BMI	Number/Sex	Symptom Duration	Outcome Measures
Chao [23] 1996	Nonsurgical treatment	66	NR	37F, 12M	NR	Functional scoring system devised based on pain, limp, use of assistive device, distance of ambulation, and patient satisfaction
Chen [24] 1997	Imaging	52	NR	11F, 3M	8 months	Sonographic findings
Hsu [25] 1997	Imaging	55	23.4	11F, 5M	NR	Sonographic findings
Lim [26] 1997	Imaging	51	NR	16F, 8M	NR	MRI findings
Premkumar [27] 2002	Imaging	43	NR	25F, 6M	NR	Ultrasound and MRI findings
Perry [28] 2003	Imaging	43	NR	25F, 6M	NR	Ultrasound and MRI findings
Wacker [29] 2003	Imaging	57	NR	10F, 2M	40 months	MRI findings
Alvarez [30] 2006	Nonsurgical treatment	50	26.3	37F, 10M	135 days	VAS for pain, orthotic use, strength
Tome [31] 2006	Gait analysis	56.8	33.7	12F, 2M	NR	Foot kinematics
Cooper [32] 2007	Diagnosis	45.3	NR	9F, 6M	10 months	Response to local anesthetic injections and its correlation with MRI findings
Ringleb [33] 2007	Gait analysis	69	29	5F	NR	Hindfoot and midfoot kinematics, plantar foot pressures and electromyographic activity of the posterior tibialis, gastrocnemius, anterior tibialis and the peroneals
Neville [34] 2007	Gait analysis	56.1	33.2	14F, 3M	NR	Changes in posterior tibialis muscle length; Short Musculoskeletal Function Assessment Index and Mobility subscale
Houck [35] 2008	Evaluation	61	30	18F, 6M	NR	Deep compartment muscle strength
Jeong [36] 2008	Nonsurgical treatment	52.5, 53.2	22.6/24.0	7F, 5F	>3 months	Pain, ROM, muscle power, AOFAS, 5 min walking test
Krause [37] 2008	Nonsurgical treatment	64.2	NR	14F, 4M	29 months	AOFAS hindfoot score, clinical and radiographic progression
Lin [38] 2008	Nonsurgical treatment	57.6	NR	27F, 5M	19.3 months	AOFAS ankle/hindfoot score, SF-36, FFI, VAS, a custom questionnaire to evaluate the results of bracing, SLHR, ROM of the subtalar joint
Houck [39] 2009	Evaluation	59.8	29.9	21F, 9M	NR	Foot kinematics during a bilateral heel rise test
Houck [40] 2009	Gait-analysis	59.3	29.6	22F, 8M	NR	Ankle and foot kinematics during stance
Kohls-Gatzoulis [41] 2009	Prevalence	>40	NR	1000F	NR	Prevalence
Kulig [42] 2009	Nonsurgical treatment	52.1	29.6	9F, 1M	At least 3 months	FFI, PAS, GRS, 5 min walk test, SLHR testtendon’s morphology through ultrasound
Kulig [43] 2009	Nonsurgical treatment	51.3/55.3/49.4	28.7/32/28.5	8F, 4M/10F, 2M/10F, 2M	25.3/26/40.5 months	FFI, 5 min walk test, VAS
Neville [44] 2009	Gait analysis	52.7	34.4	10F	NR	Foot kinematics
Chung [45] 2010	Epidemiology/Nonsurgical treatment	52.6	23.9–27.0	42F	NR	VAS, radiological findings
Neville [46] 2010	Gait analysisEvaluation	57.9/56.5	30.4/30.8	10F, 4M/9F, 7M	11/10 months	Deep posterior compartment strength, foot kinematics
Kulig [47] 2011	Evaluation	52.1	29.5	17F	NR	Ankle and hip muscle performance
Rabbito [48] 2011	Gait analysis Evaluation	30.3	23.2	9F, 3M	NR	Maximum voluntary ankle invertor muscle strength, arch height index, and 3-dimensional medial longitudinal arch and rearfoot and kinematics during walking
Neville [49] 2012	Gait analysis	63.6	31.8	5F, 10M	3–23 months	Foot kinematics
Mani [50] 2013	Reported Outcomes	59.7	NR	84F, 42M	NR	Validity, reliability, and responsiveness of FAOS
Neville [51] 2013	Evaluation	57.9/56.5	30.4/30.8	10F, 4M/9F, 7M	11/10 months	Total and distributed plantar loading and foot kinematics
Chimenti [52] 2014	Evaluation	57.6	30	14F, 6M	NR	Foot and ankle kinematics during SLHR test
Sanhudo [53] 2014	Risk factor	62	28.58	103F, 15M	NR	Frequency, mean absolute, and mean relative leg length discrepancy
Arnoldner [54] 2015	Imaging	50	NR	13F, 10M	NR	Evaluation for tendinosis, partial tear, and complete tear
Houck [55] 2015	Nonsurgical treatment	57/58	30/31	15F, 4M/13F, 4M	NR	FFI, Short musculoskeletal function assessment, isometric deep posterior compartment strength
Kulig [56] 2015	Evaluation	54.6	28.9	19F	NR	Unipedal standing balance test and SLHR
Neville [57] 2016	Gait analysis	60.1	31.3	8F, 7M	3–23 months	Foot kinematics
Heng [58] 2018	Evaluation	29.5	22.9	13F, 3M	NR	First ray mobility
Gonzalez [59] 2019	Imaging	55	NR	28F, 20M	NR	MRI-based geometric measurements
Kwon [60] 2020	Imaging	51.5	NR	16F, 5M	NR	Weight-bearing foot radiograph and sonographic findings
Park [61] 2020	Imaging	38.6	NR	5F, 9M	NR	Posterior tibial tendon cross-sectional area on MRI
Robinson [62] 2020	Nonsurgical treatment	34	NR	7F, 3M	7.5 months	FAAM ADL and Sport subscales
Chicoine [63] 2021	Gait analysis	47.6	29	11F, 3M	26.1 months	Gait biomechanics (kinetics and kinematics)
Kim [64] 2021	Nonsurgical treatment	22.2	22.6	7F, 8M	NR	Ankle joint kinematics and kinetics and tibialis anterior and fibularis longus muscle activation during gait, pain (VAS)
Koltak [65] 2021	Nonsurgical treatment	24/22	25/23.9	26/26 (NR)	NR	FFI, IPAQ, muscle strength
Ross [18] 2021	Diagnosis	46.2	30.1	42F, 10M	>3 months	Pain on tendon palpation, swelling around the tendon, pain/weakness with tibialis posterior contraction, and pain during or inability to perform a single-leg heel raise; ultrasound findings

Abbreviations: ADL, activities of daily living; AOFAS, American Orthopaedic Foot and Ankle Society; BMI, body mass index; GRS, Global Rating Scale; F, female; FAAM, Foot and Ankle Ability Measure; FAOS, The Foot and Ankle Outcome Score; FFI, Foot Functional Index; IPAQ, International Physical Activity Questionnaire; M, male; MRI, magnetic resonance imaging; NR, not reported; PAS, Physical Activity Scale; ROM, range of motion; SLHR, single-limb heel-rise; SF-36, 36-Item Short Form Survey. Footnote: Age and symptom duration expressed in mean or median depending on how the articles reported. BMI was calculated manually if the articles provided height and weight.

**Table 3:** Summary of findings from studies on non-surgical treatments.

Author (Year)	Study Design	Intervention	Clinical Outcome	Adverse Event
Chao [23] (1996)	Retrospective chart review	MAFO or UCBL depending on the foot deformity and BMI	67% of patients reported good to excellent results based on pain, function, use of assistive device, ambulating distance, and satisfaction. Four patients (out of 49) underwent surgery after tolerating orthosis poorly.	Nine patients stopped wearing the orthosis due to discomfort and inconvenience
Alvarez [30] (2006)	Prospective, observational study	Orthosis, exercise, and stretching	Over a median period of 4 months after a median of 10 physical therapy sessions, 83% had successful subjective and functional outcomes. A total of 89% of patients were satisfied; the remining 11% of patients required surgery after failing conservative management.	Not reported
Jeong [36] (2008)	RCT	Stretching and strengthening versus control	The stretching and strengthening group demonstrated significant improvement in pain, dorsiflexion ROM, and dorsiflexion and plantarflexion power compared to controls.	Not reported
Krause [37] (2008)	Prospective case series	Custom-molded foot orthosis (Shell brace)	AOFAS hindfoot scored significantly improved during the follow-up, and 15 out 18 patients (83%) reported pain relief within 2.8 weeks. A total of 83% of patients did not show progression with the treatment over a mean follow-up of 5 years.	Adverse events were seen in three patients (3/18: 16%) who showed development of calluses.
Lin [38] (2008)	Retrospective chart review	Double Upright Ankle Foot Orthosis	The study found that 69.7% of the patients were brace-free and able to avoid surgery at an average follow-up of 8.6 years with significant improvement in AOFAS hindfoot score, FFI, and VAS pain score.	Not reported
Kulig [42] (2009)	Prospective case series	Eccentric loading and stretching with orthoses	A significant improvement was noticed in FFI scores, the number of single heel rise, and global rating scale, but tendon morphology and vascularization remained abnormal at 6 months.	Not reported
Kulig [43] (2009)	RCT	orthoses + stretching (O group) versus orthoses + stretching + concentric versus (OC group) orthoses + stretching + eccentric (OE group)	All groups showed significant improvement in FFI, but the OE group demonstrated the most improvement in each subcategory while the O group showed the least. Pain immediately after the 5 min walk test was also significantly decreased across the groups.	Not reported
Chung [45] (2010)	Retrospective chart review	Modified protocol by Alvarez et al.	VAS pain scores significantly improved at the mean follow-up of 20 months, and 28 of 39 patients reported improvements in single heel rise. Five patients elected to complete surgical treatment.	Not reported
Houck [55] (2015)	RCT	Strengthening (AirLift orthosis + stretching + strengthening—progressive loading including eccentric and concentric exercises) versus stretching (AirLift orthosis + stretching)	Both groups showed significant improvement in pain and function without minimal difference between the groups over the 12-week period. Additionally, there was no difference in isometric deep posterior compartment strength.	No adverse event
Robinson [62] (2020)	Retrospective case series	Radial ESWT and foot core exercise	Clinically important differences in the FAAM were seen in 9 patients (out of 10) for ADL and 8 patients for sport subscales.	No adverse event
Kim [64] (2021)	Prospective pre-post study	Short-foot exercise program	There were positive effects on modifying muscle activation patterns for tibialis anterior and fibularis longus without influencing structural deformity and ankle joint moments. Additionally, no significant improvement was observed in pain measured by VAS.	Not reported
Koltak [65] (2021)	RCT	Insole versus LLLT	FFI scores improved significantly in both groups after treatments, but the insole group had significantly better results at 9 months. Additionally, physical activity levels increased significantly in both groups, but those of the insole group were significantly higher during the follow-up.	Not reported

Abbreviation: ADL, activities of daily living; AOFAS, American Orthopaedic Foot and Ankle Society; BMI, body mass index; ESWT, extracorporeal shockwave therapy; FFI, foot functional index; LLLT, low level light therapy; MAFO, molded ankle-foot orthosis; RCT, randomized controlled trial; UCBL, University of California Biomechanics Laboratory; VAS; visual analogue scale.

## Data Availability

Not applicable.
